# Disentangling craving‐ and valence‐related brain responses to smoking cues in individuals with nicotine use disorder

**DOI:** 10.1111/adb.13083

**Published:** 2021-08-07

**Authors:** Amelie Haugg, Andrei Manoliu, Ronald Sladky, Lea M. Hulka, Matthias Kirschner, Annette B. Brühl, Erich Seifritz, Boris B. Quednow, Marcus Herdener, Frank Scharnowski

**Affiliations:** ^1^ Psychiatric University Hospital Zurich Zurich Switzerland; ^2^ Faculty of Psychology University of Vienna Vienna Austria; ^3^ McLean Hospital Belmont Massachusetts USA; ^4^ Harvard Medical School Harvard University Boston Massachusetts USA; ^5^ Montreal Neurological Institute McGill University Montreal Canada

**Keywords:** craving, cue‐reactivity, functional Magnetic Resonance Imaging, neuroimaging, nicotine use disorder, smoking

## Abstract

Tobacco smoking is one of the leading causes of preventable death and disease worldwide. Most smokers want to quit, but relapse rates are high. To improve current smoking cessation treatments, a better understanding of the underlying mechanisms of nicotine dependence and related craving behaviour is needed. Studies on cue‐driven cigarette craving have been a particularly useful tool for investigating the neural mechanisms of drug craving. Here, functional neuroimaging studies in humans have identified a core network of craving‐related brain responses to smoking cues that comprises of amygdala, anterior cingulate cortex, orbitofrontal cortex, posterior cingulate cortex and ventral striatum. However, most functional Magnetic Resonance Imaging (fMRI) cue‐reactivity studies do not adjust their stimuli for emotional valence, a factor assumed to confound craving‐related brain responses to smoking cues. Here, we investigated the influence of emotional valence on key addiction brain areas by disentangling craving‐ and valence‐related brain responses with parametric modulators in 32 smokers. For one of the suggested key regions for addiction, the amygdala, we observed significantly stronger brain responses to the valence aspect of the presented images than to the craving aspect. Our results emphasize the need for carefully selecting stimulus material for cue‐reactivity paradigms, in particular with respect to emotional valence. Further, they can help designing future research on teasing apart the diverse psychological dimensions that comprise nicotine dependence and, therefore, can lead to a more precise mapping of craving‐associated brain areas, an important step towards more tailored smoking cessation treatments.

## Graphical abstract

Most fMRI‐based cue‐reactivity studies do not adjust their stimuli for emotional valence, a factor assumed to confound craving‐related brain responses to smoking cues. Here, we investigated the influence of emotional valence on key addiction brain areas by disentangling craving‐ and valence‐related brain responses with parametric modulators. For the amygdala, we observed significantly stronger brain responses to the valence aspect of the presented images than to the craving aspect, emphasizing the need for carefully selecting stimulus material for cue‐reactivity paradigms.

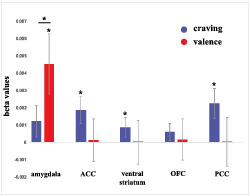

## 1 INTRODUCTION

Tobacco smoking is one of the leading risk factors for preventable death and disease worldwide, and it is estimated to kill 8 million users per year.^1^, ^2^ Even though the number of nicotine‐dependent smokers who want to quit smoking is large,^3^ many smokers are not successful with their smoking cessation goals, and relapse rates are high.^4^ In addition, the efficacy of available psychological and pharmacological treatments against nicotine dependence is still limited.^5^ Causes for smoking relapse are diverse, and, to date, a wide range of factors that have an effect on relapse rates have been identified, such as perceived stress,^6^ impulsivity,^7^ or low self‐efficacy.^8^

Another important factor that influences success in quitting to smoke is the individual's smoking‐related craving^9^ and the ability to control this craving.^10^, ^11^ Craving, or, more precisely, drug craving, is commonly described as the ‘desire to use a drug’,^12^ even though precise operational definitions can still vary largely across studies.^13^ In particular, Shiffman and colleagues^14^ observed cigarette craving after waking up to be predictive for smoking relapse in smokers who wanted to quit. Consequently, encountering social situations associated with smoking and other craving‐inducing environments pose serious problems for smokers to reach their goal of sustained abstinence. Therefore, cigarette craving has been intensively studied with different methods.^13^ For example, self‐report questionnaires, which can vary from a single visual analogue scale^15^ to more complex, multi‐dimensional questionnaires such as the Questionnaire on Smoking Urges,^29^ have been established as suitable methods to assess baseline craving levels. Another important approach that focuses more on situationally induced craving is the so‐called cue‐reactivity paradigm.^16^, ^17^ Here, smokers are exposed to smoking cues, such as real cigarettes or visual stimuli that depict smoking scenes, and their subjective, behavioural or physiological craving response is measured. Cue‐reactivity paradigms combined with neuroimaging are particularly suitable for identifying human brain responses associated with craving.^18^, ^19^, ^20^

**FIGURE 1:**
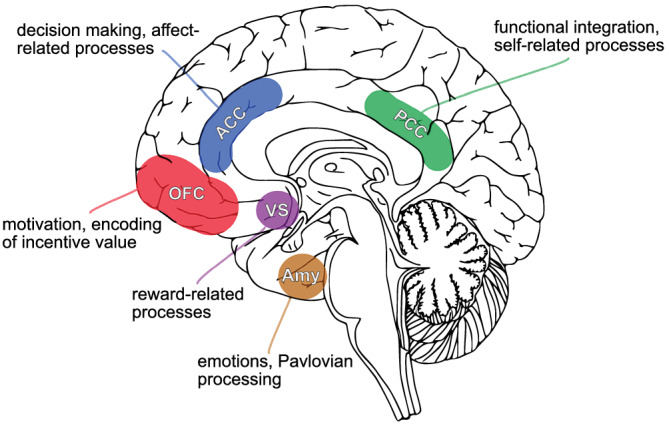
Most commonly observed brain areas in fMRI‐based cue‐reactivity studies. Cue‐reactivity meta‐analyses^18^, ^19^, ^20^ identified a wide range of different brain areas driven by drug cues, with the amygdala (Amy), the ventral striatum (VS), the anterior cingulate cortex (ACC), the orbitofrontal cortex (OFC) and the posterior cingulate cortex being the most robustly identified cue‐driven brain areas

Recent meta‐analyses of cue‐reactivity functional Magnetic Resonance Imaging (fMRI) studies found relatively robust drug cue‐related brain activity in the ventral striatum, amygdala, anterior cingulate cortex (ACC), posterior cingulate cortex (PCC) and orbitofrontal cortex (OFC)^18^, ^19^, ^20^ (see Figure 1). These brain areas were repeatedly identified in cue‐reactivity studies and may play an important role in the neural mechanisms underlying substance use disorders.^30^ For instance, the ventral striatum is considered as one of the most prominent reward‐associated areas and has been directly linked to reward processing and reward learning in animal and human studies,^21^ making it a key area involved in addiction. Also, the amygdala, next to its strong involvement in emotional processes,^22^ also mediates Pavlovian processing, making it a crucial instance in the learning of associations between environmental cues and drug‐induced reward and, therefore, in reinforcing drug seeking behaviour.^23^ The ACC, PCC and OFC, known to be involved in higher order cognitive processes, have also been linked to neural mechanisms related to substance use disorder: the ACC as a particularly strongly connected brain area is associated with decision‐making as well as executive control and exerts top‐down control over reward‐related areas such as the ventral striatum.^24^ The OFC is involved in evaluating the value of rewards and, therefore, another important area for reward processing.^25^ Finally, the PCC has been observed to be more indirectly involved in substance use disorder. The PCC is part of the default mode network and associated with self‐related processes, being activated when resisting cue‐driven craving.^26^

Cue‐reactive brain responses have also been shown to be predictive of smoking abstinence and relapse,^27^, ^28^ thus further underlining the clinical importance of understanding the neural mechanisms in these brain areas regarding nicotine dependence and craving.

The multifaceted neural underpinnings of smoking cue‐reactivity reflect the many psychological dimensions that are associated with the perception and processing of such cues. Smoking cues are associated with the urge to smoke as well as factors such as the positive or negative connotation of the depicted situation. Consequently, the unique contribution of smoking‐related craving to measured neural responses can be confounded by other stimulus dimensions that, as well, influence addiction‐related brain areas. In particular, the emotional dimension of smoking cues can potentially confound craving‐associated responses, as affective stimuli have been shown to strongly engage brain areas like the amygdala,^31^ an area that has also been identified in a wide range of cue‐reactivity studies.^18^, ^19^, ^20^ This is of particular relevance as the majority of fMRI‐based cue‐reactivity studies (e.g., 80% of the fMRI studies analysed in the aforementioned three meta‐analyses) used a classical fMRI block design to identify brain areas that are related to smoking‐associated cues: blocks of drug‐related images are presented in an alternating fashion with blocks of neutral images. An fMRI block design provides considerably large statistical power to detect effects, but its inflexible design of a priori selected images for each stimulus block does not allow for further analyses with respect to other stimulus characteristics.^32^ This inflexibility can, consequently, be problematic when other stimulus characteristics (other than the ones chosen for designing the stimulus blocks) are driving different/additional brain dynamics. For instance, craving‐related brain regions might also be related to other aspects of the presented smoking cues, such as their emotional content.

To overcome this limitation, we applied an event‐related parametric fMRI design combined with ratings of craving and valence as behavioural measures to disentangle core components of craving‐ and valence‐related brain regions. This allowed us to identify key regions within the ‘addiction network’^30^ that were more sensitive to and more driven by either the craving or the emotional content of the presented craving cues. A better understanding of specific craving—as well as valence‐related brain activations—can help to optimize future cue‐reactivity paradigms and, more importantly, can help to identify more reliable targets for clinical interventions, such as cue‐exposure therapy or real‐time fMRI neurofeedback.

## 2 MATERIAL AND METHODS

### 2.1 Participants

Thirty‐two subjects with nicotine use disorder (age: 25.93 ± 5.30; 16 females, 15 males and 1 non‐binary; average daily cigarette consumption of 11.47 ± 5.57 cigarettes; smoking duration of 7.41 ± 4.76 years) participated in the study. This sample size is based on power calculations using previous studies included in cue‐reactivity meta‐analyses^18^, ^19^, ^20^ with maximally 31 subjects that have been able to reject the null hypothesis in one or more voxels (α‐error probability of 5% adjusted for multiple comparisons). All subjects gave informed written consent and were compensated for their participation in the study (25CHF/h). Exclusion criteria were mental or neurological disorders, MRI‐incompatibility criteria (i.e., metal implants, current pregnancy and pacemakers), as well as the use of any non‐cigarette tobacco substitutes, such as nicotine patches, chewing gums or electronic cigarettes. Inclusion criterium was tobacco use disorder according to DSM‐5.

### 2.2 Experimental procedure and design

All subjects were instructed to abstain from smoking at least 1 h before the study. Prior to scanning, subjects were asked to fill out several questionnaires which included a drug use anamnesis of current and past drug use (previously described in Quednow et al.^33^), the Fagerström Test for Nicotine Dependence^34^ and the brief Questionnaire of Smoking Urges (QSU).^35^ The QSU was filled out a second time in the end of the experimental session.

**FIGURE 2:**
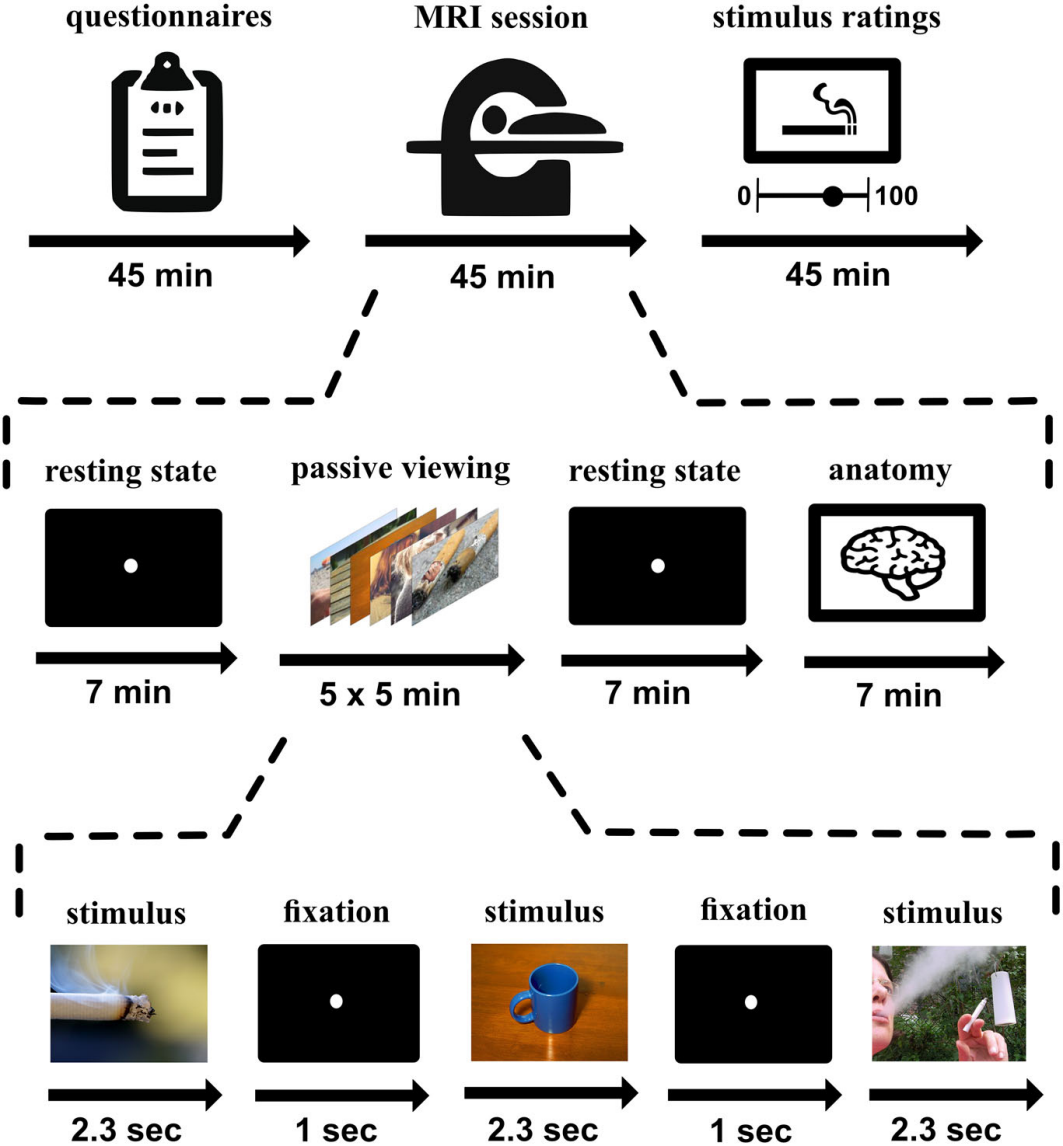
Experimental design. The study was divided into three parts. In the first part, subjects filled out several questionnaires on their smoking routines. Then, subjects underwent 50 min of scanning, including resting state scans, five runs of passive viewing and an anatomical scan. During each passive viewing run, 68 out of a total of 340 neutral or nicotine‐related images (including 10 additional catch trial images) were presented for 2.3 s in random order, followed by a 1‐s fixation dot baseline. Finally, subjects rated all 330 presented images with respect to craving and valence

In the MRI session, subjects underwent a passive viewing paradigm (code for image presentation available at https://osf.io/6y8fu/) where they were presented a total of 330 neutral and nicotine‐related images, distributed over five runs of 4 min each (see Figure 2). During each run, 68 images were presented for 2.3 s in random order, each followed by a 1‐s fixation dot as baseline. In the beginning of each run, a 15‐s fixation dot baseline was presented. To ensure that all images were passively viewed by the subjects and subjects were paying attention to the images, 10 additional catch trial images were randomly presented during the five runs.

Participants were instructed to perform a button press when a catch trial image, depicting an exclamation mark, was presented. All images were taken from the Smoking Cue Database (SmoCuDa),^36^ the International Affective Picture System (IAPS) database,^37^ and the International Smoking Image Series (ISIS) database^38^ and covered a continuous range from very mild to very intense craving‐inducing content. To control for habituation effects, all images were presented only once and in randomized order in the MR scanner. Before and after the passive viewing runs, participants also performed 7‐min resting state scans where they had to fixate a white fixation dot over a black background. After the functional imaging, we acquired an anatomical image. In total, one scanning session took approximately 50 min.

After scanning, subjects were asked to rate the 330 images that were presented during the passive viewing paradigm on a 100‐point visual analogue scale outside the scanner. All images were presented in random order and had to be rated on two dimensions: the subject's urge to smoke when seeing the image (craving) and how positively or negatively the subject perceived the image (valence). A detailed description of the rating procedure can be found in Manoliu et al.^36^

### 2.3 MRI data acquisition

MR images were acquired with a 3 Tesla Philips Achieva scanner (Philips Healthcare, the Netherlands) using a 32‐channel head coil at the MR Center of the Psychiatric University Hospital in Zurich, Switzerland. Functional images during the passive viewing paradigm were acquired using a T2*‐weighted gradient‐echo planar imaging (EPI) sequence with repetition time (RT) = 2,000 ms, echo time (TE) = 35 ms, flip angle (FA) = 82°, 27 slices in ascending order, interslice gap = 1 mm, voxel size = 2 × 2 × 3 mm^3^ and field of view (FoV) = 220 × 220 × 109 mm^3^. Five dummy scans as well as 122 functional images were collected during each 4‐min passive viewing run. Further, a T1‐weighted sequence in the end of the session was acquired with FA = 8°, 237 sagittal slices in ascending order, no slice gap, voxel size = 0.76 × 0.76 × 0.76 and FoV = 255 × 255 × 180 mm^3^. The anatomical run took 8:26 min.

### 2.4 fMRI analysis

All functional MR images were analysed using MATLAB2017a and Statistical Parametric Mapping (SPM12; Wellcome Trust Centre for Neuroimaging, London, United Kingdom). Preprocessing of the functional images included slice‐time correction, realignment, co‐registration of the functional scans to the anatomical image, segmentation, normalization into Montreal Neurological Institute (MNI) space and smoothing with a Gaussian kernel of 6 mm full width at half maximum.

Before the parametric analysis, we applied a more standard cue‐reactivity analysis without parametric modulation to replicate previous approaches. For this, we first specified a general linear model (GLM) with three regressors for high and low craving‐intensity smoking stimuli as well as neutral stimuli and included the six motion parameters as well as the catch trials as regressors of no interest. We then contrasted smoking images that were rated as highly craving inducing, defined as images that received a craving rating of 50 or higher, with neutral images (high‐craving‐vs.‐neutral contrast). Overall, 60 stimuli were defined as neutral, 129 stimuli were defined as high‐craving and 141 stimuli were defined as low‐craving stimuli, based on mean group ratings.

For our parametric analyses, we specified two GLMs, GLM‐craving and GLM‐valence. Each GLM contained the six motion parameters as regressors of no interest and one regressor representing the presented images. GLM craving additionally contained one parametric modulator for the craving rating for each presented stimulus, while GLM valence additionally contained one parametric modulator for the valence rating for each presented stimulus. In both GLMs, catch trials were modelled as an additional regressor of no interest. Contrast images representing the craving and valence regressor, respectively, were used for second level analyses.

For the region of interest (ROI)‐based analysis, we extracted average values from the beta images of the craving and valence parametric regressor of the first level analyses, for each ROI, using custom MATLAB scripts (all used scripts can be found on the Open Science Framework: https://osf.io/6y8fu/). The ROIs were defined based on fMRI cue‐reactivity meta studies^18^, ^19^, ^20^ and included the ventral striatum, amygdala, orbitofrontal cortex, anterior cingulate cortex and posterior cingulate cortex (see the supporting information for a detailed description of ROI selection and definition and the Open Science Framework repository for the mask files: https://osf.io/6y8fu/). One meta‐analysis did not differentiate between cue‐reactivity studies on different substance use disorders^18^; for the other two meta‐analyses, we focused on results based on smoking cue‐reactivity only. We compared the extracted craving‐ and valence‐beta values using paired *t* tests. To investigate brain‐behaviour relationships, we performed Spearman correlation analyses between extracted beta values and Fagerström dependence scores as well as the number of daily smoked cigarettes, respectively.

### 2.5 Behavioral analysis

Ratings of the parametric modulators were based on average ratings across 40 nicotine‐dependent subjects, including the 32 participants of this study, and were performed after the scanning session. A detailed description of the rating procedure and rating distributions has been published in Manoliu et al.^36^

### 2.6 Statistical analyses

All statistical cluster‐level analyses of fMRI data were performed using an initial cluster defining threshold of *p* < 0.001 and family‐wise‐error (FWE) corrections of 0.05. Paired *t* tests between craving and valence betas were corrected for multiple comparisons using Bonferroni corrections.

### 2.7 Code and data availability

The used smoking stimuli, a more detailed overview of stimuli ratings and the exact scripts used for performing stimulus ratings are all available on the Open Science Framework (OSF) platform (https://osf.io/6gwy5/). Further, we have made all scripts used for fMRI analyses, and image presentation is publicly available in another repository on the OSF platform (https://osf.io/6y8fu/). This repository also includes group‐level neuroimaging data, while individual data were not allowed to be shared publicly by our local ethics regulations.

## 3 RESULTS

### 3.1 Brain response to high‐craving smoking images as compared to neutral images

**FIGURE 3:**
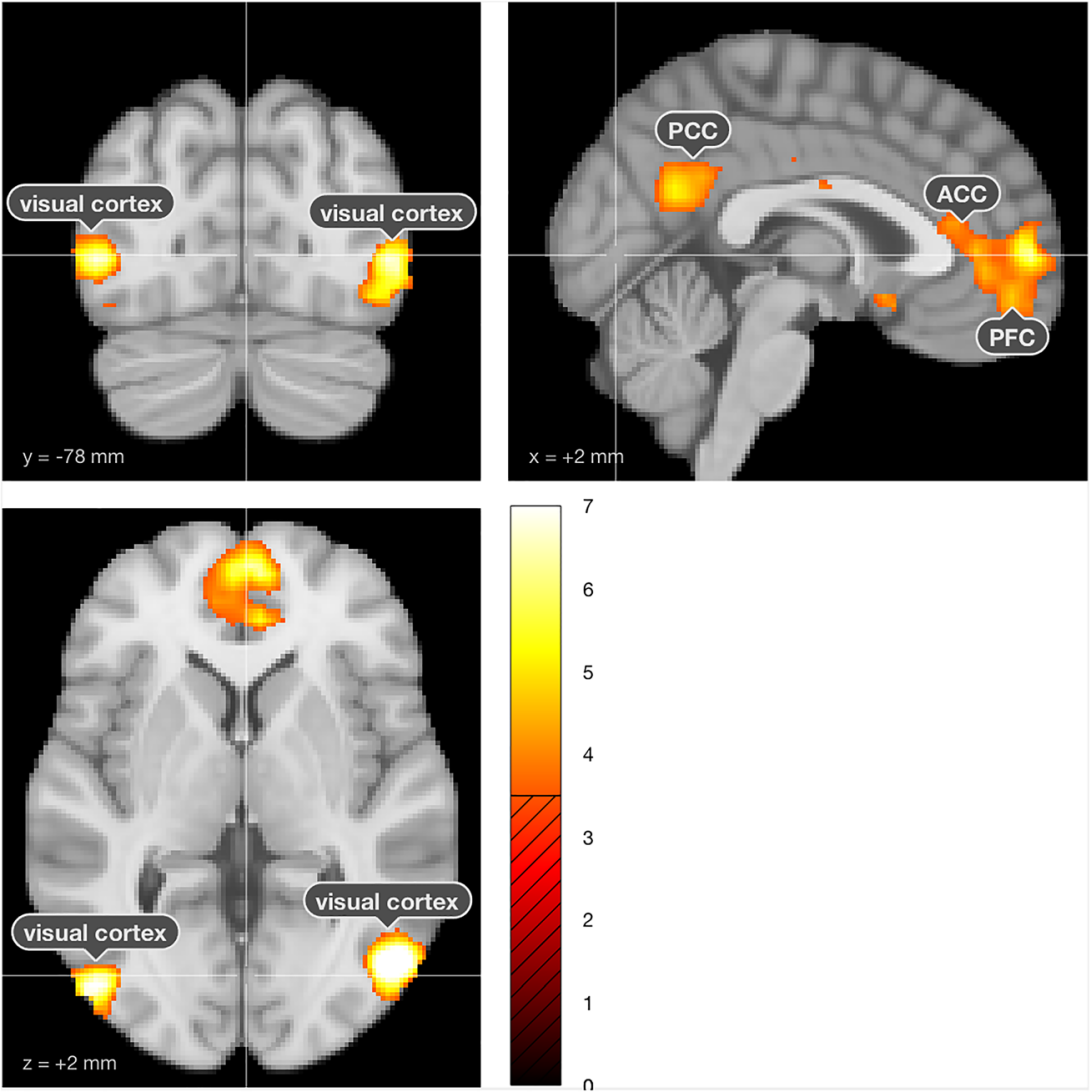
Whole‐brain analysis results depicting brain areas activated by high‐craving smoking images as contrasted to neutral images. The high‐craving‐versus‐neutral contrast revealed activation in the higher order visual cortices, the prefrontal cortex (PFC) and anterior cingulate cortex (ACC) and the posterior cingulate cortex (PCC)

To replicate conventional cue‐reactivity analyses, we contrasted high‐craving‐versus‐neutral, depicting brain areas related to high‐in‐craving‐rated smoking images as compared to neutral images. Our analysis revealed significant (0.05 FWE‐corrected) activation in the higher order visual cortices, the medial prefrontal cortex and ACC, and the PCC (see Figure 3 and Table S1 for details). For completeness, we report the respective results for a low‐craving‐versus‐neutral contrast in the supporting information (Figure S1 and Table S2). Finally, we found significant small‐volume corrected activation for the ACC, OFC and PCC, but not the amygdala and ventral striatum (Table S6).

### 3.2 Disentangling craving and valence activations

To disentangle brain activations associated specifically with craving or valence, we parametrically analysed responses to the craving and valence rating, respectively. Whole brain analyses revealed significant craving‐related activation within the visual cortex, the PFC and ACC, the PCC, the parietal cortex, the fusiform gyrus and the precuneus, while significant valence‐related activation was observed for the visual cortex and the parietal cortex. More details can be found in the supporting information (Figures S2 and S3; Tables S3 and S4).

**FIGURE 4:**
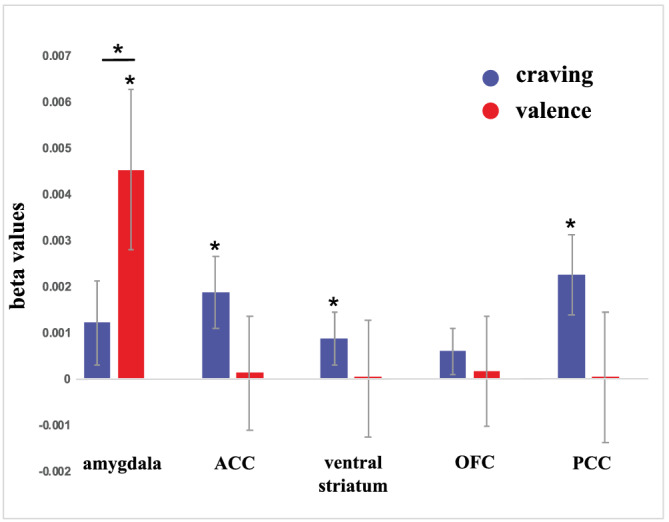
Brain responses to the craving and valence ratings of the presented images. Group‐level analyses revealed significant craving‐related activation within the ACC, ventral striatum, and PCC and significant valence‐related activation within the amygdala. We observed a significant difference between craving‐ and valence‐related activation within the amygdala. Abbreviations: anterior cingulate cortex (ACC), orbitofrontal cortex (OFC) and posterior cingulate cortex (PCC)

In a second step, we specifically investigated craving‐ and valence‐associated activity within our five predefined ROIs, namely ACC, amygdala, OFC, PCC and ventral striatum. The craving regressor showed significant small volume‐corrected activation in the OFC, the ACC, the ventral striatum and the PCC (Figure 4; Table S5). In contrast, the valence regressor only showed significant small volume‐corrected activation within the amygdala. Statistical comparisons using paired *t* tests showed a significant difference between the craving‐ and valence‐related responses in the amygdala, with valence‐related amygdala activity being significantly higher than craving‐related activity (*t*[31] = 4.41, *p* < 0.001; Figure 4). The other four investigated ROIs did not show significant differences between craving‐ and valence‐related brain responses. Nevertheless, in these four ROIs, craving‐related brain responses were higher than valence‐related brain responses with the latter often reaching values close to zero (see Figure 4). Further details can be found in Table S5.

## 4 DISCUSSION

Nicotine use disorder is functionally multidimensional, and so is the neural response to smoking‐related cues. Here, we disentangled brain responses to two core dimensions associated with smoking‐related cues: craving and valence. Using a parametric fMRI design, we investigated the influence of craving and valence aspects on key addiction brain areas: the amygdala, the ventral striatum, the ACC, the PCC and the OFC. Our approach enables a more precise mapping of craving‐associated brain areas.

Our results show that the chosen brain areas that are robustly recruited by smoking cues in meta‐analyses^18^, ^19^, ^20^ were indeed activated by the craving‐aspect of the presented cues (Figure 4). Here, it should be noted that smoking cue reactivity in single studies can be dependent on context (e.g., drug availability right after the scanning session) and the subject population (e.g., treatment seeking subjects vs. non‐treatment seeking subjects) and, therefore, might differ from large‐scale meta‐analyses.^39^

However, when isolating the emotional content of the smoking cues and its influence on neural responses within these ROIs, we observed that amygdala activation is related to the images' valence ratings. This valence‐related response in the amygdala was significantly stronger than the craving‐related amygdala response. In contrast, the ACC, ventral striatum, OFC and PCC were not (strongly) affected by the emotional content of the presented images. Hence, the amygdala responds primarily to the valence dimension of smoking cues, which is a novel finding in the cue‐reactivity literature. However, it should be noted that, even though we did not observe significantly stronger valence‐related neural responses in the ACC, ventral striatum, OFC and PCC, we also did not observe significantly stronger craving‐related responses in these areas. This might be due to a lack of statistical power.

Our findings have practical implications for the interpretation of previous studies, the design of future cue‐reactivity research and for the further development of neuronal models of addiction. The amygdala has frequently been reported as activated in smoking cue‐reactivity paradigms and has correctly been identified as an important region in addiction.^23^, ^40^, ^41^ However, previous studies did not disentangle which dimension the amygdala responds to. According to a literature search of the studies included in three cue‐reactivity meta‐analyses,^18^, ^19^, ^20^ only two out of 49 studies^42^, ^43^, that is, only 4% of the studies, accounted for stimulus valence in their study designs. Further, 80% of the included studies used a cue‐reactivity block‐design with the other studies using an event‐related, yet non‐parametric design. In neuroimaging, classical block designs are commonly used for because of their greater statistical power compared to, for example, parametric designs.^32^, ^44^ However, when blocks of craving cues are contrasted with blocks of neutral images, the resulting brain activations reflect differences between these craving cues and neutral images along all functional dimensions of the stimuli and of nicotine dependence. Our results show that when the influence of valence is not accounted for in a cue‐reactivity design, amygdala responses will most likely be driven by the valence and not the craving aspect of the presented smoking cues. This can be avoided by, for example, adjusting for valence during stimulus selection or specifically focusing on the craving dimension using a parametric modulator analysis approach. Publicly available smoking cue databases such as SmoCuDa (https://smocuda.github.io/ ^36^) provide suitable stimuli for implementing such designs. If this is not possible, the uncertainty regarding which smoking dimension is causing the activations should be kept in mind when interpreting them. These suggestions do not just apply to smoking research but can also inform other fields that work with fMRI‐based cue reactivity paradigms, such as alcohol use disorder,^45^ heroin use disorder,^46^ cocaine use disorder,^47^ obesity^48^ and gambling disorder.^49^

We applied a parametric fMRI design combined with behavioural craving and valence ratings to disentangle core components of craving‐ and valence‐related brain regions. Such a specific parametric cue‐reactivity approach can grant more detailed and precise insights into the complex dynamics of cue‐driven craving in the brain, as it allows for focusing on separable stimulus dimension. Using this design, we identified key regions within the addiction network that were more sensitive to and more driven by either the craving or the emotional content of the presented smoking cues. Future studies might also investigate further stimulus dimensions which might be intertwined with craving‐related results in key brain areas for addiction, such as salience or arousal. This can help to further specify the functional role of brain regions included in existing neuronal models of addiction.^24^, ^50^, ^51^ A better functional understanding and more precise mapping of the neural underpinnings of craving might also help to identify more reliable targets for clinical interventions, such as cue‐exposure therapy or real‐time fMRI neurofeedback. In particular, novel brain‐based treatment approaches such as neurofeedback rely strongly on the correct selection of target brain areas and signals.^52^ Neurofeedback has been shown to be a promising tool for treating dysfunctional brain signals in substance use disorder,^53^, ^54^ and in the field of tobacco use disorder, several brain areas have been successfully trained to reduce smoking cue‐driven drug craving and to support smoking cessation.^55^, ^56^, ^57^

## 5 CONCLUSION

Using a parametric cue‐reactivity paradigm, we disentangled brain responses to craving and valence dimensions of smoking cues. Our findings suggest that the amygdala responds primarily to the valence component of such cues. This can refine the interpretation of previous reports of amygdala activity during smoking cue‐reactivity and help designing future research aimed at teasing apart the many psychological dimensions that comprise nicotine dependence. Also, a more precise mapping of craving‐related brain areas is an important step towards more tailored smoking cessation treatments.

## CONFLICT OF INTEREST

No support by the industry was received. All authors declare no conflict of interest.

## ETHICS STATEMENT

This study was conducted in accordance with the Declaration of Helsinki and was approved by the local ethics committee (University of Zurich).

## AUTHORS CONTRIBUTIONS

AH, AM, RS, LMH, MK, ABB, BBQ, MH and FS were responsible for the conceptualization of the project. AH and AM were responsible for data curation. AH was responsible for the formal analysis. AH, AM, RS, MK, BBQ, MH and FS were responsible for the methodology. RS provided software. LMH, ABB, ES, MH and FS provided resources. FS was provided funding and supervision. AH, AM and FS created a first draft of the manuscript. All authors critically reviewed the content of the manuscript and approved the final version for publication.

## Supporting information


**Table S1:** **Whole‐brain analysis results depicting brain areas activated by high‐craving smoking images as contrasted to neutral images; p < 0.05 FWE‐corrected.** Overview of the statistical values for all clusters and peaks. Abbreviations: PPC: posterior cingulate cortex, ACC: anterior cingulate cortex, PFC: prefrontal cortex, FWE: family‐wise error.
**Table S2: Whole‐brain analysis results depicting brain areas activated by low‐craving smoking images as contrasted to neutral images; p < 0.05 FWE‐corrected.** Overview of the statistical values for all clusters and peaks. Abbreviations: FWE: family‐wise error.
**Table S3: Whole‐brain analysis results depicting brain areas driven by the parametric modulator for craving ratings.** Overview of the statistical values for all clusters and peaks, using an initial threshold of p < 0.001 (uncorrected). Abbreviations: FWE: family‐wise error, vmPFC: ventromedial prefrontal cortex, ACC: anterior cingulate cortex, PCC: posterior cingulate cortex.
**Table S4: Whole‐brain analysis results depicting brain areas driven by the parametric modulator for valence ratings.** Overview of the statistical values for all clusters and peaks, using a p < 0.05 FWE‐correction. Abbreviations: FWE: family‐wise error.
**Table S5: Brain responses to the craving and valence ratings of the presented images.** All small volume corrections were cluster‐level family wise error corrected. Mean beta values depict beta values based on group‐level analyses. SVC: small volume correction.
**Table S6: Brain responses to the craving and valence ratings of the presented images.** All small volume corrections were cluster‐level family wise error corrected. Mean beta values depict beta values based on group‐level analyses. SVC: small volume correction.
**Figure S1: Brain areas activated by low‐craving images as contrasted to neutral images.** Please see Table S2 for a detailed list of all significant clusters, including the visual cortex, the prefrontal cortex, the left middle temporal gyrus, and the right superior frontal gyrus.
**Figure S2: Brain areas driven by the craving ratings of the presented stimuli.** Please see Table S3 for a detailed list of all significant clusters, including the visual cortices, the ventromedial prefrontal cortex and anterior cingulate cortex, the posterior cingulate cortex, the right parietal cortex, the left fusiform gyrus, and the precuneus.
**Figure S3: Brain areas driven by the valence ratings of the presented stimuli.** Please see Table S4 for a detailed list of all significant clusters, including the visual cortices, and the parietal cortices.

## Data Availability

Code and data availability The used smoking stimuli, a more detailed overview of stimuli ratings, and the exact scripts used for performing stimulus ratings are all available on the Open Science Framework (OSF) platform (https://osf.io/6gwy5/). Further, we have made all scripts used for fMRI analyses and image presentation publicly available in another repository on the OSF platform (https://osf.io/6y8fu/). This repository also includes group‐level neuroimaging data, while individual data were not allowed to be shared publicly by our local ethics regulations.
