# Supplementary material for: Disentangling craving‐ and valence‐related brain responses to smoking cues in individuals with nicotine use disorder
Source: Addict Biol. 2021 Aug 7;27(1):e13083. doi: 10.1111/adb.13083 (PMC9285426; doi:10.1111/adb.13083)
Supplement: Supplementary file 1 — Table S1: Whole‐brain analysis results depicting brain areas activated by high‐craving smoking images as contrasted to neutral images; p < 0.05 FWE‐corrected. Overview of the statistical values for all clusters and peaks. Abbreviations: PPC: posterior cingulate cortex, ACC: anterior cingulate cortex, PFC: prefrontal cortex, FWE: family‐wise error. Table S2: Whole‐brain analysis results depicting brain areas activated by low‐craving smoking images as contrasted to neutral images; p < 0.05 FWE‐corrected. Overview of the statistical values for all clusters and peaks. Abbreviations: FWE: family‐wise error. Table S3: Whole‐brain analysis results depicting brain areas driven by the parametric modulator for craving ratings. Overview of the statistical values for all clusters and peaks, using an initial threshold of p < 0.001 (uncorrected). Abbreviations: FWE: family‐wise error, vmPFC: ventromedial prefrontal cortex, ACC: anterior cingulate cortex, PCC: posterior cingulate cortex. Table S4: Whole‐brain analysis results depicting brain areas driven by the parametric modulator for valence ratings. Overview of the statistical values for all clusters and peaks, using a p < 0.05 FWE‐correction. Abbreviations: FWE: family‐wise error. Table S5: Brain responses to the craving and valence ratings of the presented images. All small volume corrections were cluster‐level family wise error corrected. Mean beta values depict beta values based on group‐level analyses. SVC: small volume correction. Table S6: Brain responses to the craving and valence ratings of the presented images. All small volume corrections were cluster‐level family wise error corrected. Mean beta values depict beta values based on group‐level analyses. SVC: small volume correction. Figure S1: Brain areas activated by low‐craving images as contrasted to neutral images. Please see Table S2 for a detailed list of all significant clusters, including the visual cortex, the prefrontal cortex, the left middle temporal gyrus, [file ADB-27-0-s001.docx]

**SUPPLEMENTAL MATERIAL**

**Title** Disentangling craving- and valence-driven brain responses to smoking cues

**Authors** Amelie Haugg*, Andrei Manoliu*, Ronald Sladky, Lea M. Hulka, Matthias Kirschner, Annette Brühl, Erich Seifritz, Boris B. Quednow, Marcus Herdener, Frank Scharnowski

*^*^ AH and AM contributed equally to this work.*

*Region of interest selection*

To identify key ROIs of the addiction network, we aggregated information from meta analyses on fMRI-based cue-reactivity studies that described a contrast between drug cues and neutral cues. In total, three recent meta analyses were taken into consideration: Meta analysis 1 (MA1) by Chase an colleagues (Chase, Eickhoff, Laird, & Hogarth, 2011), meta analysis 2 (MA2) by Kühn and colleagues (Kühn & Gallinat, 2011), and meta analysis 3 (MA3) by Tang and colleagues (Tang, Fellows, Small, & Dagher, 2012). ROIs that were mentioned in at least two of these meta analyses were considered for further analyses in this study: the ventral striatum (MA1, MA2, MA3), the amygdala (MA1, MA2, MA3), the orbitofrontal cortex (OFC; MA1, MA3), the posterior cingulate cortex (PCC; MA1, MA3), and the anterior cingulate cortex (ACC; MA2, MA3). Masks for the ROIs (except for the OFC) were taken from WFU Pickatlas (<https://www.nitrc.org/projects/wfu_pickatlas/>). The OFC mask was defined as a 10mm sphere with a center at (4, 46, -9; taken from MA1) as no WFU Pickatlas mask was available for the OFC. The defined masks contained 469 (amygdala), 2713 (ACC), 127 (NAcc), 515 (OFC), and 798 (PCC) voxels.

*Brain responses to high-craving smoking images as compared to neutral images*

|  | cluster | peak | peak | peak | coordinates | | | anatomy |
| --- | --- | --- | --- | --- | --- | --- | --- | --- |
| p(FWE-corr) | **equivk** | **p(FWE-corr)** | **T** | **equivZ** | **x** | **y** | **z {mm}** |  |
| 0 | 293 | 0 | 9.56 | 6.48 | 51 | -70 | 2 | visual cortex |
| 0 | 184 | 0.001 | 7.22 | 5.49 | -48 | -79 | 2 | visual cortex |
|  |  | 0.62 | 4.72 | 4.07 | -51 | -67 | 11 |  |
|  |  | 0.905 | 4.32 | 3.79 | -60 | -64 | 17 |  |
| 0 | 566 | 0.006 | 6.6 | 5.18 | 3 | 59 | 5 | PFC/ACC |
|  |  | 0.103 | 5.62 | 4.63 | -9 | 53 | -4 |  |
|  |  | 0.166 | 5.45 | 4.53 | 6 | 41 | 2 |  |
|  |  | 0.662 | 4.67 | 4.03 | -6 | 38 | 5 |  |
|  |  | 0.86 | 4.4 | 3.85 | 6 | 59 | -13 |  |
|  |  | 0.923 | 4.28 | 3.76 | 0 | 35 | 14 |  |
|  |  | 0.953 | 4.2 | 3.7 | 21 | 47 | -10 |  |
|  |  | 0.964 | 4.15 | 3.68 | 9 | 68 | -7 |  |
|  |  | 0.996 | 3.9 | 3.49 | -21 | 38 | -10 |  |
|  |  | 0.999 | 3.82 | 3.43 | -3 | 26 | 20 |  |
|  |  | 0.999 | 3.8 | 3.42 | -12 | 74 | -1 |  |
|  |  | 1 | 3.64 | 3.3 | -15 | 44 | -13 |  |
| 0 | 170 | 0.136 | 5.52 | 4.57 | 3 | -58 | 26 | PCC |
|  |  | 1 | 3.73 | 3.37 | -3 | -64 | 17 |  |
| 0.002 | 80 | 0.508 | 4.86 | 4.16 | 24 | 26 | 50 | PFC |
|  |  | 0.66 | 4.67 | 4.04 | 18 | 38 | 41 |  |
|  |  | 0.914 | 4.3 | 3.78 | 21 | 44 | 50 |  |

**Table S1: Whole-brain analysis results depicting brain areas activated by high-craving smoking images as contrasted to neutral images; p < 0.05 FWE-corrected.** Overview of the statistical values for all clusters and peaks. Abbreviations: PPC: posterior cingulate cortex, ACC: anterior cingulate cortex, PFC: prefrontal cortex, FWE: family-wise error.

*Brain responses to low-craving smoking images as compared to neutral images*


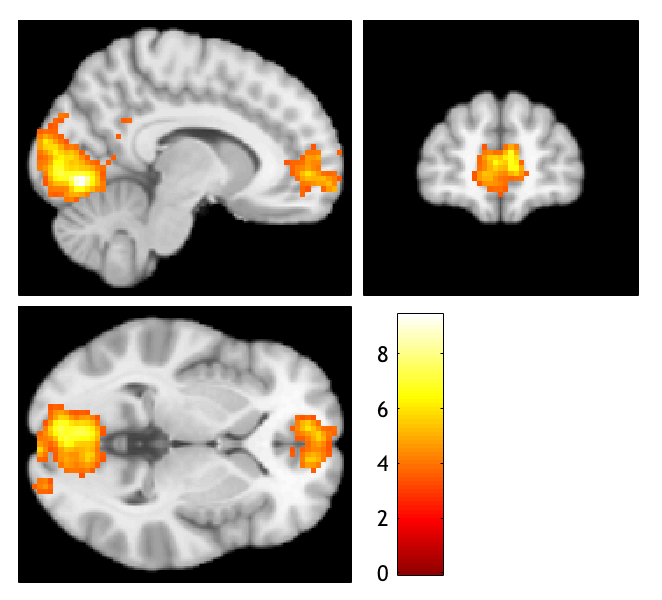


**Figure S1: Brain areas activated by low-craving images as contrasted to neutral images.** Please see Table S2 for a detailed list of all significant clusters, including the visual cortex, the prefrontal cortex, the left middle temporal gyrus, and the right superior frontal gyrus.

| **cluster** | | **peak** | | | **coordinates** | | | **anatomy** |
| --- | --- | --- | --- | --- | --- | --- | --- | --- |
| **p(FWE-corr)** | **equivk** | **p(FWE-corr)** | **T** | **equivZ** | **x** | **y** | **z** |  |
| 0.000 | 1759 | 0.000 | 9.42 | 6.43 | -12 | -76 | -7 | visual cortex |
|  |  | 0.001 | 7.27 | 5.52 | -9 | -88 | -1 |  |
|  |  | 0.001 | 7.24 | 5.50 | -3 | -73 | 2 |  |
|  |  | 0.002 | 6.96 | 5.36 | -3 | -100 | 11 |  |
|  |  | 0.011 | 6.42 | 5.08 | -6 | -91 | 11 |  |
|  |  | 0.044 | 5.91 | 4.80 | 9 | -88 | 5 |  |
| 0.000 | 357 | 0.000 | 7.64 | 5.69 | 6 | 59 | 8 | prefrontal cortex |
|  |  | 0.011 | 6.41 | 5.08 | -3 | 56 | 5 |  |
|  |  | 0.050 | 5.88 | 4.78 | -9 | 53 | -1 |  |
| 0.014 | 61 | 0.134 | 5.49 | 4.56 | -63 | -16 | -13 | left middle temporal gyrus |
| 0.005 | 75 | 0.844 | 4.37 | 3.83 | 18 | 35 | 41 | right superior frontal gyrus |

**Table S2: Whole-brain analysis results depicting brain areas activated by low-craving smoking images as contrasted to neutral images; p < 0.05 FWE-corrected.** Overview of the statistical values for all clusters and peaks. Abbreviations: FWE: family-wise error.

*Whole brain analyses of the craving parametric modulator*


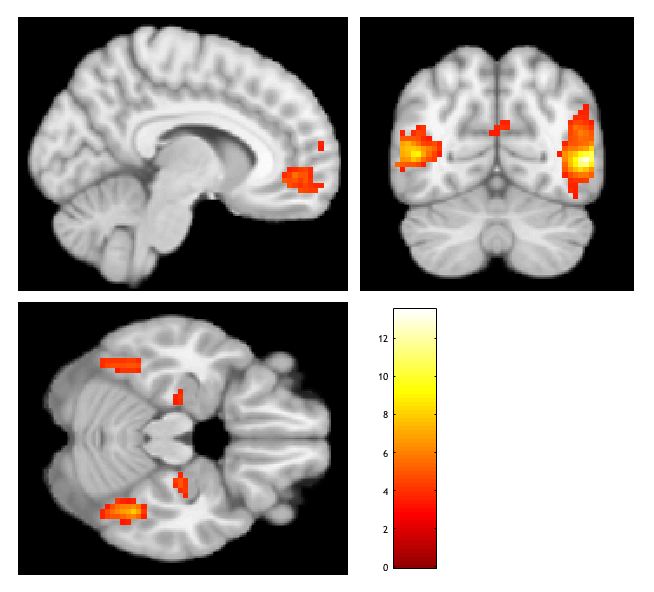


**Figure S2: Brain areas driven by the craving ratings of the presented stimuli.** Please see Table S3 for a detailed list of all significant clusters, including the visual cortices, the ventromedial prefrontal cortex and anterior cingulate cortex, the posterior cingulate cortex, the right parietal cortex, the left fusiform gyrus, and the precuneus.

| **cluster** | | **peak** | | **coordinates** | | | **anatomy** |
| --- | --- | --- | --- | --- | --- | --- | --- |
| p(FWE-corr) | **equivk** | **p(FWE-corr)** | **T** | **x** | **y** | **z** |  |
| 0 | 585 | 0 | 13.48 | 48 | -73 | 2 | Right visual cortex |
|  |  | 0 | 8.15 | 42 | -82 | -7 |  |
|  |  | 0 | 7.73 | 42 | -46 | -19 |  |
|  |  | 0.006 | 6.5 | 45 | -64 | 23 |  |
| 0 | 379 | 0 | 12.92 | -45 | -79 | 2 | Left visual cortex |
| 0 | 404 | 0.003 | 6.7 | -3 | 50 | -13 | vmPFC/ACC |
|  |  | 0.004 | 6.66 | 3 | 41 | 5 |  |
|  |  | 0.009 | 6.37 | -3 | 41 | -7 |  |
|  |  | 0.011 | 6.28 | 6 | 56 | -13 |  |
|  |  | 0.036 | 5.88 | 9 | 44 | -10 |  |
| 0 | 99 | 0.053 | 5.74 | 3 | -61 | 26 | PCC |
| 0.023 | 47 | 0.299 | 5.01 | 27 | -43 | 50 | Right parietal cortex |
| 0.015 | 52 | 0.315 | 4.99 | -42 | -46 | -16 | Left fusiform gyrus |
|  |  | 0.64 | 4.55 | -42 | -58 | -19 |  |
| 0.019 | 49 | 0.659 | 4.53 | 0 | -55 | 53 | precuneus |
|  |  | 0.85 | 4.27 | 3 | -49 | 47 |  |

**Table S3: Whole-brain analysis results depicting brain areas driven by the parametric modulator for craving ratings.** Overview of the statistical values for all clusters and peaks, using an initial threshold of p < 0.001 (uncorrected). Abbreviations: FWE: family-wise error, vmPFC: ventromedial prefrontal cortex, ACC: anterior cingulate cortex, PCC: posterior cingulate cortex.

*Whole brain analyses of the valence parametric modulator*


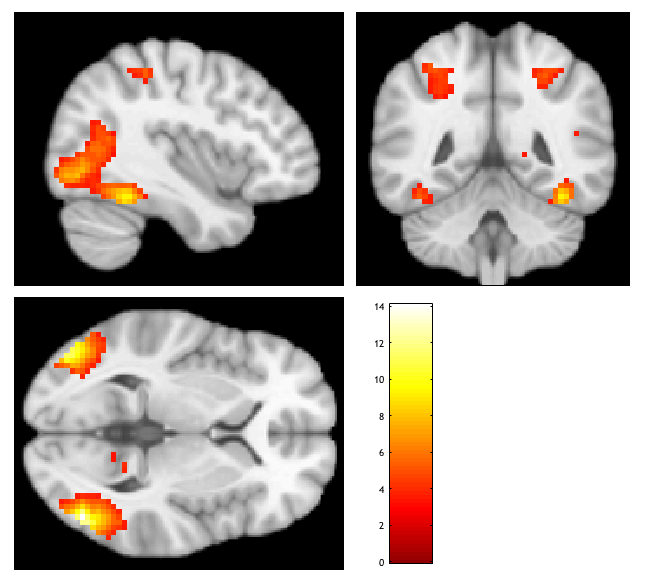


**Figure S3: Brain areas driven by the valence ratings of the presented stimuli.** Please see Table S4 for a detailed list of all significant clusters, including the visual cortices, and the parietal cortices.

| **cluster** | | **peak** | | **coordinates** | | | **anatomy** |
| --- | --- | --- | --- | --- | --- | --- | --- |
| **p(FWE-corr)** | **equivk** | **p(FWE-corr)** | **T** | **x** | **y** | **z** |  |
| 0 | 665 | 0 | 14.07 | 48 | -73 | 2 | Right visual cortex |
|  |  | 0 | 9.23 | 39 | -49 | -19 |  |
| 0 | 463 | 0 | 11.01 | -45 | -82 | 2 | Left visual cortex |
|  |  | 0 | 9.32 | -48 | -70 | 5 |  |
|  |  | 0.068 | 5.65 | -42 | -43 | -19 |  |
| 0.001 | 96 | 0.004 | 6.62 | 27 | -43 | 50 | Right parietal cortex |
|  |  | 0.018 | 6.12 | 36 | -37 | 50 |  |
| 0 | 114 | 0.058 | 5.71 | -30 | -43 | 50 | Left parietal cortex |

**Table S4: Whole-brain analysis results depicting brain areas driven by the parametric modulator for valence ratings.** Overview of the statistical values for all clusters and peaks, using a p < 0.05 FWE-correction. Abbreviations: FWE: family-wise error.

|  | **craving** | | **valence** | |
| --- | --- | --- | --- | --- |
| **Region of interest** | **Mean beta value** | **p-value SVC** | **Mean beta value** | **p-value SVC** |
| **Amygdala** | 0.0014 | No supra-threshold cluster | 0.0049 | 0.009 |
| **Anterior cingulate cortex** | 0.0022 | 0.000 | -0.000225 | No supra-threshold cluster |
| **Ventral striatum** | 0.000994 | 0.018 | 0.000119 | No supra-threshold cluster |
| **Orbitofrontal cortex** | 0.000622 | No supra-threshold cluster | 0.000037 | No supra-threshold cluster |
| **Posterior cingulate cortex** | 0.0023 | 0.002 | 0.000104 | No supra-threshold cluster |

**Table S5: Brain responses to the craving and valence ratings of the presented images.** All small volume corrections were cluster-level family wise error corrected. Mean beta values depict beta values based on group-level analyses. SVC: small volume correction.

|  | **High craving versus neutral** | |
| --- | --- | --- |
| **Region of interest** | **Mean beta value** | **p-value SVC** |
| **Amygdala** | 0.0081 | No suprathreshold clusters |
| **Anterior cingulate cortex** | 0.1958 | 0.000 |
| **Ventral striatum** | 0.0476 | No suprathreshold clusters |
| **Orbitofrontal cortex** | 0.4032 | 0.000 |
| **Posterior cingulate cortex** | 0.1342 | 0.000 |

**Table S6: Brain responses to the craving and valence ratings of the presented images.** All small volume corrections were cluster-level family wise error corrected. Mean beta values depict beta values based on group-level analyses. SVC: small volume correction.
